# Drug susceptibility testing of clinical isolates of streptococci and enterococci by the Phoenix automated microbiology system

**DOI:** 10.1186/1471-2180-7-46

**Published:** 2007-05-23

**Authors:** Gioconda R Brigante, Francesco A Luzzaro, Beatrice Pini, Gianluigi Lombardi, Gertrude Sokeng, Antonio Q Toniolo

**Affiliations:** 1 Laboratorio di Microbiologia, Università dell'Insubria e Ospedale di Circolo, I-21100 Varese, Italy

## Abstract

**Background:**

Drug resistance is an emerging problem among streptococcal and enterococcal species. Automated diagnostic systems for species identification and antimicrobial susceptibility testing (AST) have become recently available. We evaluated drug susceptibility of clinical isolates of streptococci and enterococci using the recent Phoenix system (BD, Sparks, MD). Diagnostic tools included the new SMIC/ID-2 panel for streptococci, and the PMIC/ID-14 for enterococci. Two-hundred and fifty isolates have been investigated: β-hemolytic streptococci (n = 65), *Streptococcus pneumoniae *(n = 50), viridans group streptococci (n = 32), *Enterococcus faecium *(n = 40), *Enterococcus faecalis *(n = 43), other catalase-negative cocci (n = 20). When needed, species ID was determined using molecular methods. Test bacterial strains were chosen among those carrying clinically-relevant resistance determinants (penicillin, macrolides, fluoroquinolones, glycopeptides). AST results of the Phoenix system were compared to minimal inhibitory concentration (MIC) values measured by the Etest method (AB Biodisk, Solna, Sweden).

**Results:**

Streptococci: essential agreement (EA) and categorical agreement (CA) were 91.9% and 98.8%, respectively. Major (ME) and minor errors (mE) accounted for 0.1% and 1.1% of isolates, respectively. No very major errors (VME) were produced. Enterococci: EA was 97%, CA 96%. Small numbers of VME (0.9%), ME (1.4%) and mE (2.8%) were obtained. Overall, EA and CA rates for most drugs were above 90% for both genera. A few VME were found: a) teicoplanin and high-level streptomycin for *E. faecalis*, b) high-level gentamicin for *E. faecium*. The mean time to results (± SD) was 11.8 ± 0.9 h, with minor differences between streptococci and enterococci.

**Conclusion:**

The Phoenix system emerged as an effective tool for quantitative AST. Panels based on dilution tests provided rapid and accurate MIC values with regard to clinically-relevant streptococcal and enterococcal species.

## Background

Resistance of streptococci and enterococci to different classes of antimicrobials is increasing worldwide [1,2]. Reduced susceptibility or resistance to penicillin and other β-lactams have been reported with particular frequency in the case of *Streptococcus pneumoniae *and viridans streptococci [1,3]. Resistance to macrolides and fluoroquinolones has been described in viridans and β-hemolytic streptococci [4,5]. Notably, glycopeptide resistance is an increasing threat among enterococcal species, especially *Enterococcus faecium *and *E. faecalis *[2].

Since clinical isolates of all the above species may produce severe infections (sepsis, meningitis, endocarditis), rapid and accurate identification (ID) and antimicrobial susceptibility testing (AST) are of great significance. For these bacteria, evaluation of drug susceptibility using disk diffusion methods can be problematic since the technique does not provide quantitative values. In this context, the Clinical Laboratory Standards Institute (CLSI; formerly NCCLS) established that *S. pneumoniae *isolates should not be reported as penicillin-resistant or intermediate based solely on an oxacillin zone ≤ 19 mm [6]. For these strains, minimum inhibitory concentration (MIC) values of penicillin and cefotaxime/ceftriaxone or meropenem are mandatory. Correct detection of vancomycin resistance among enterococci is also critical, since diffusion is difficult for large molecules and interpretative criteria are based on limited differences in the inhibition zone. MIC values may be of further assistance in discriminating enterococcal species carrying different *van*-type determinants.

Reproducibility of results, accuracy of species ID, precision of quantitative AST results, turnaround time, availability of data for epidemiological monitoring, and cost-effectiveness represent compelling reasons that support the choice of an automated system for clinical bacteriology. Systems of this type are now available for catalase-negative gram-positive cocci [7-9]. In the year 2000, Becton Dickinson (BD Diagnostic Systems, Sparks, MD) introduced the Phoenix Automated Microbiology System for ID and AST of gram-negative bacilli, staphylococci, and enterococci [10-13]. More recently, a panel for streptococcal species (SMIC/ID-2 panel) has been added [14,15].

The present study was designed to evaluate the performance of the Phoenix system for the quantitative determination of drug susceptibility of streptococcal and enterococcal isolates carrying different resistance determinants. Time to results was also analyzed.

## Results

Bacterial isolates are listed in Table 1. AST results are expressed as raw data (i.e., as results not interpreted by the expert system). For streptococcal isolates (Table 2), the overall essential agreement (EA; 91.9%) and categorical agreement (CA; 98.8%) between the Phoenix system and the Etest method were satisfactory. No VME were produced. Very low rates of major errors (ME; 0.1%) and minor errors (mE; 1.1%) were found. For enterococcal isolates (Table 3), the overall EA and CA were 97% and 96%, respectively. Small numbers of errors were found: VME (0.9%), ME (1.4%), mE (2.8%).

As shown in Tables 2 and 3, the overall performance for different antimicrobials was rather satisfactory (mean EA for streptococci ≥ 91%, for enterococci ≥ 97%). Poor performances were, however, noticed for streptococci in the case of clindamycin (EA, 67.3%) and erythromycin (EA, 88.6%), and for enterococci in the case of teicoplanin (EA, 87%). CA rates ≥ 97% and ≥ 94% were obtained for streptococci and enterococci, respectively. However, the low CA rate obtained in the case of linezolid for enterococci (89%) should be noticed.

Table 4 shows the AST results relative to different species or groups of streptococci. Mean CA values were as follows: *S. pneumoniae *95%, β-hemolytic-streptococci 99.4%, and viridans streptococci 99.6%. With regard to beta-lactams, mE have been obtained only with *S. pneumoniae*: penicillin (12%), cefotaxime (2%), and cefepime (2%). Among *S. pyogenes *isolates, one ME for clindamycin was observed. As shown in Table 5, rather satisfactory CA values were obtained with *E. faecalis *(98.2%) and *E. faecium *(96.7%). Occasional VME have been produced for teicoplanin and HL-streptomycin in *E. faecalis*, and for HL-gentamicin in *E. faecium*. Due to the reduced numbers, results of isolates belonging to species other than *E. faecalis *and *E. faecium *(see Table 1) are not presented in Table 5. For these species, mean CA value was 88.9%; mE and ME were 7.2% and 5.3%, respectively. Errors were restricted to chloramphenicol, tetracycline, linezolid, and quinupristin-dalfopristin.

### Time to results (TTR)

TTR values represent the overall time required for both ID and AST results. The mean TTR ± SD was 11.8 ± 0.9 h, with minor differences between streptococci (12.4 ± 2.2) and enterococci (11.4 ± 3.0). Among streptococci, β-hemolytic species showed the lowest TTR (*S. agalactiae*, 11.3 h; *S. pyogenes*, 11.6 h; *S. dysgalactiae*, 11.7 h). *S. pneumoniae *and viridans group streptococci were characterized by longer TTR (13.1 h and 13.3 h, respectively). Among enterococci, TTR was 9.9 ± 2.8 h for *E. faecalis*, and 11.7 ± 2.3 h for *E. faecium*.

## Discussion

Over 70 streptococcal and enterococcal species have been implicated in animal and/or human infections [16,17]. Of these, only a few cause important human infections (e.g., *S. pyogenes*, *S. pneumoniae, S. agalactiae*, *E. faecalis, E. faecium*, viridans streptococci).

Streptococcal and enterococcal isolates characterized by a wide spectrum of drug susceptibility have been investigated to assess the performance of the Phoenix system using both the new SMIC/ID-2 panel for streptococci and the established PMIC/ID-14 panel for enterococci. Both panels allowed testing clinically-relevant drugs: penicillins, third- and fourth-generation cephalosporins, macrolides, fluoroquinolones, glycopeptides, and the newer molecules linezolid and quinupristin-dalfopristin.

The results demonstrate the overall satisfactory performance of the Phoenix system. CA and EA values were consistently > 90% (98.8% and 91.9%, for streptococci; 96% and 97%, for enterococci). The lowest CA values were obtained with *S. pneumoniae *and with enterococci other than *E. faecalis *and *E. faecium*.

For streptococci, results in agreement with ours have been obtained by Japanese investigators [14,15] that used SMIC/ID panels differing from those used by us. As compared to the automated Vitek system, the Phoenix appeared more accurate in determining MIC values for pneumococci [18]. Excellent results were obtained with both systems with regard to *S. agalactiae *[9]. Among enterococci, our results showed lower performances with respect to what has been reported by others using both the Phoenix and the Vitek systems [9,12,19]. In this respect, it should be noted that a high proportion (46/100) of our isolates carried glycopeptide resistance determinants and that 17/100 isolates were of species other than *E. faecalis *and *E. faecium*. Problems with AST results for the above species have also been reported by others using automated systems [20].

Unsatisfactory results have been obtained for clindamycin in the case of streptococci (EA, 67.3%): MIC values produced by the Phoenix were, in fact, usually lower than those obtained with the Etest. Investigated strains, however, showed MICs consistently within breakpoint values, thus limiting the clinical impact of the error. The reduced response to clindamycin was not related to the expression of resistance determinants (inducible/constitutive MLS_B _phenotype, or efflux; data not shown). Overall, small numbers of VME and ME were obtained. Most errors were classified as mE, due to minor differences in MIC values around breakpoint concentrations. Regarding ten enterococcal isolates, the Phoenix system gave MIC values of 4 μg/ml for linezolid, thus classifying the isolates as "intermediate". MIC values given by the Etest were instead of 2 μg/ml (i.e., "susceptible"). The finding appears to reflect a problem already noticed by Phoenix users with regard to this drug. Thus, increased linezolid MICs need to be verified using a reference method. Regarding penicillin, mE were encountered in 6/150 streptococci. In the latter cases, MICs were overestimated by the Phoenix system. Overall, the results of the present study were superior to those previously reported for both the Phoenix and the VITEK 2 system [14,15,18].

A mE rate < 10% is recommended for accepting AST results, and up to 3.0% ME and 1.5% VME are acceptable [21]. Overall, the results produced by the Phoenix were well within the recommended range. However, an ME rate of 5.3% was obtained for enterococci other than *E. faecium *and *E. faecalis*.

## Conclusion

The Phoenix system appears an effective diagnostic tool for infections caused by streptococci and enterococci. Panels based on dilution tests were able to deliver adequate quantitative AST results in short times. This is especially relevant for severe infections caused by aggressive species such as *S. pyogenes*, *S. pneumoniae, E. faecalis*, and *E. faecium*. For these organisms, interpretation of susceptibility data varies according to the infection site and – in the case of large molecules that hardly diffuse in solid media (e.g., glycopeptides) – results obtained using dilution methods are to be preferred [20,22].

## Methods

### Clinical isolates

Bacterial strains were obtained from routine clinical specimens at the Microbiology Laboratory of the Ospedale di Circolo, Varese (Italy). A total of 250 non-duplicate clinical isolates of gram-positive, catalase-negative cocci were studied. When needed, species ID was obtained by molecular methods [23]. The following species were investigated (Table 1): *S. pneumoniae*, *Streptococcus pyogenes*, *Streptococcus agalactiae*, *Streptococcus dysgalactiae *subsp. *equisimilis*, viridans group streptococci, *E. faecium*, *E. faecalis*, plus other streptococcal and enterococcal species. Test organisms included isolates characterized by different susceptibility levels to penicillin, macrolides, and fluoroquinolones. Among enterococci (n = 100), vancomycin resistance determinants were present in 46 isolates: *vanA *(33 isolates), *vanB *(2 isolates), *vanC1 *(7 isolates), and *vanC2 *(4 isolates). Fifty-four vancomycin-susceptible isolates were shown not to carry *van*-type determinants. Glycopeptide-resistance determinants (*vanA*, *vanB*, *vanC-1*, *vanC-2*, *van-C-3*, *vanD*, *vanE*, and *vanG*) were investigated by PCR following reported methods [24]. Isolates were stored at -70°C in Todd-Hewitt broth containing 20% glycerol. Before performing ID and AST assays, the isolates were passed twice on Mueller-Hinton agar containing 5% sheep blood (Oxoid SpA, Milan, Italy) to get them to an active growth stage following metabolic inactivity while frozen.

### Phoenix AST

The Phoenix system uses two different panels for ID and AST of gram-positive cocci: the SMIC/ID-2 panel for streptococci, and the PMIC/ID-14 panel for enterococci and staphylococci. Panels have two separate sections. The left one contains ID substrates. The AST section (right side) contains three to eight concentrations of different drugs. Panel inoculation has been performed according to the manufacturer's instructions. As recommended, the same ID broth has been used for both panel types. Two different broth types are instead needed for AST. The AST-S broth containing growth supplements plus one drop of the Phoenix AST-S indicator is intended for streptococci. The AST broth plus one drop of the Phoenix AST indicator is intended for enterococci, staphylococci, and gram-negative bacteria.

Bacterial isolates have been obtained from fresh overnight cultures, suspended in the ID broth and adjusted to the 0.5 McFarland standard using a dedicated nephelometer (CrystalSpec, BD). Twenty-five microliters of the ID suspension have been used to inoculate AST broths, resulting in the final inoculum density of approximately 5 × 10^5 ^CFU/ml. Inoculated AST broths have been poured into the appropriate sections of each panel. Panels have been sealed, logged, and loaded into the instrument at 35°C. Kinetic, colorimetric and fluorescent signals have been collected every 20 min until ID and AST results were completed.

### Comparator AST method

MICs have been determined by the Etest method (AB Biodisk, Solna, Sweden) according to manufacturer's instructions (preparation of inoculum, plating, strip application, reading of results). Streptococcal isolates have been cultured on Mueller-Hinton agar plates supplemented with 5% sheep blood (Oxoid). Enterococcal isolates have been tested on Mueller-Hinton agar plates (BD). Vancomycin has been tested on brain heart infusion plates (Oxoid). MIC values have been interpreted according to CLSI criteria [6]. Different drug panels have been tested for streptococci and enterococci: a) streptococci (penicillin, erythromycin, clindamycin, cefotaxime, cefepime, levofloxacin, vancomycin, chloramphenicol); b) enterococci [ampicillin, vancomycin, teicoplanin, high-level (HL) streptomycin, HL gentamicin, chloramphenicol, quinupristin-dalfopristin, tetracycline, linezolid].

### Analysis of data and discrepancies

AST results are reported as raw data, i.e. without interpretation by the expert system. Results have been categorized as susceptible (S), intermediate (I), or resistant (R) according to CLSI [6]. MIC values produced by the Phoenix method (TEST) have been compared to those obtained with the Etest method (COMP). The following definitions have been adopted: 1) EA (i.e., MIC values of TEST panel equal to or within ± 1 dilution of the COMP value); 2) CA (i.e., TEST and COMP MIC values agree using insterpretative CLSI criteria); 3) mE (i.e., COMP is S or R and TEST is I; alternatively, COMP is I and TEST is S or R); 4) ME (i.e., COMP is S and TEST is R; the percentage of major errors has been calculated only for susceptible isolates); 5) VME (i.e., COMP is R and TEST is S; the percentage of very major errors has been calculated only for resistant isolates). In the case of ME or VME, the organism has been re-tested with both the Phoenix and the Etest. When differences persisted, the results have been considered discordant.

### Quality controls

As controls, the following strains have been included in each run: *S. pneumoniae *ATCC 49619, *S. agalactiae *ATCC 13813, and *E. faecalis *ATCC 29212.

## Abbreviations

Antimicrobial susceptibility testing (AST); Categorical agreement (CA); Clinical Laboratory Standards Institute (CLSI); Comparator method (COMP); Essential agreement (EA); Identification (ID); Intermediate (I); Major errors (ME); Minimal inhibitory concentration (MIC); Minor errors (mE); Resistant (R); Susceptible (S); Test method (TEST); Time to results (TTR); Very major errors (VME).

## Authors' contributions

GRB and BP carried out drug susceptibility tests and species identification; FAL and GL provided bacterial isolates together with clinical data; GS carried out molecular studies; AQT conceived and coordinated the study. All authors read and approved the final manuscript.

## Figures and Tables

**Table 1:** Streptococcal and enterococcal isolates investigated in the study.

Organism	No. isolates
Streptococci (n = 150)	
*Streptococcus pneumoniae*	50
Beta-hemolytic streptococci (n = 65)	
*Streptococcus pyogenes*	20
*Streptococcus agalactiae*	20
*Streptococcus dysgalactiae *subsp. *equisimilis*	25
*S. bovis*	3
Viridans streptococci (n = 32)	
*Streptococcus mitis *group (n = 10)	
*S. mitis*	5
*S. oralis*	4
*S. cristatus*	1
*Streptococcus anginosus *group (n = 11)	
*S. anginosus*	6
*S. intermedius*	2
*S. constellatus*	3
*Streptococcus sanguinis *group (n = 10)	
*S. sanguinis*	3
*S. parasanguinis*	4
*S. gordonii*	3
*Streptococcus mutans*	1
Enterococci (n = 100)	
*Enterococcus faecalis*	43
*Enterococcus faecium*	40
Other enterococcal species (n = 17)	
*Enterococcus gallinarum*	7
*Enterococcus durans*	5
*Enterococcus casseliflavus*	4
*Enterococcus avium*	1

Total	250

**Table 2:** Streptococci: overall agreement of AST results between the Phoenix system and the Etest method.

Antibiotic	Susceptibility (No.)			Essential agreement	Categorical agreement	Minor errors		Major errors	Very major errors
	
	S	I	R	%	%	No. (%)		No. (%)	No.
Penicillin	126	19	5	93.3	96.0	6	(4.0)	0	0
Erythromycin	95	0	55	88.6	100	0		0	0
Clindamycin	108	2	40	67.3	97.3	3	(2.0)	1 (0.9)	0
Levofloxacin	133	0	17	90.7	99.3	1	(0.7)	0	0
Chloramphenicol	139	0	11	97.3	99.3	1	(0.7)	0	0
Cefotaxime	149	0	1	98.0	99.3	1	(0.7)	0	0
Cefepime	145	4	1	100	99.3	1	(0.7)	0	0
Vancomycin	150	0	0	100	100	0		0	0

Total	1,045	25	130	91.9	98.8	13	(1.1)	1 (0.1)	0

**Table 3:** Enterococci: overall agreement of AST results between the Phoenix system and the Etest method.

Antibiotic	Susceptibility (No.)			Essential agreement	Categorical agreement	Minor errors		Major errors		Very major errors	
	
	S	I	R	%	%	No. (%)		No. (%)		No. (%)	
Ampicillin	62	0	38	100	100	0		0		0	
Vancomycin	59	10	31	97.0	96.0	4	(4.0)	0		0	
Teicoplanin	73	3	24	87.0	94.0	3	(3.0)	2	(2.7)	1	(4.2)
HL streptomycin	47	0	53	99.0	99.0	0		0		1	(1.9)
HL gentamicin	53	0	47	99.0	99.0	0		0		1	(2.1)
Chloramphenicol	81	3	16	95.0	94.0	2	(2.0)	4	(4.9)	0	
Linezolid	100	0	0	97.0	89.0	10	(10.0)	1	(1.0)	0	
Quinupristin-dalfopristin	36	9	55	100	94.0	6	(6.0)	0		0	
Tetracycline	50	1	49	99.0	99.0	0		1	(2.0)	0	

Total	561	26	313	97.0	96.0	25	(2.8)	8	(1.4)	3	(0.9)

**Table 4:** Agreement of AST results produced by the Phoenix system and the Etest method: streptococci of different species or groups.

Antibiotic	% of corresponding results											
	
	*S. pneumoniae *(n = 50)				Beta-hemolytic streptococci (n = 65)				Viridans group streptococci (n = 32)			
	
	CA	mE	ME	VME	CA	mE	ME	VME	CA	mE	ME	VME
Penicillin	88.0	12.0	0	0	100	0	0	0	100	0	0	0
Erythromycin	100	0	0	0	100	0	0	0	100	0	0	0
Clindamycin	96.0	4.0	0	0	98.5	0	1.7	0	96.9	3.1	0	0
Levofloxacin	100	0	0	0	98.5	1.5	0	0	100	0	0	0
Chloramphenicol	100	0	0	0	98.5	1.5	0	0	100	0	0	0
Cefotaxime	98.0	2.0	0	0	100	0	0	0	100	0	0	0
Cefepime	98.0	2.0	0	0	100	0	0	0	100	0	0	0
Vancomycin	100	0	0	0	100	0	0	0	100	0	0	0

Total	95.0	5.0	0	0	99.4	0.4	0.2	0	99.6	0.4	0	0

**Table 5:** Agreement of AST results produced by the Phoenix system and the Etest method: *E. faecalis *and *E. faecium*.

Antibiotic	% of corresponding results							
	
	*E. faecalis *(n = 43)				*E. faecium *(n = 40)			
	
	CA	mE	ME	VME	CA	mE	ME	VME
Ampicillin	100	0	0	0	100	0	0	0
Vancomycin	97.7	2.3	0	0	92.5	7.5	0	0
Teicoplanin	97.7	0	0	16.7	87.5	7.5	5.7	0
HL streptomycin	97.7	0	0	6.7	100	0	0	0
HL gentamicin	100	0	0	0	97.5	0	0	4.0
Chloramphenicol	100	0	0	0	97.5	2.5	0	0
Linezolid	90.7	9.3	0	0	97.5	2.5	0	0
Quinupristin-dalfopristin	100	0	0	0	100	0	0	0
Tetracycline	100	0	0	0	100	0	0	0

Total	98.2	1.3	0	1.4	96.7	2.5	1.0	0.7
